# Truman Capote as a Poet

**DOI:** 10.14797/mdcvj.1270

**Published:** 2023-08-01

**Authors:** James B. Young

**Affiliations:** 1 Cleveland Clinic and Professor Emeritus of Medicine, Cleveland Clinic Lerner College of Medicine of Case Western Reserve University, Cleveland, Ohio, US; 2 *Poet’s Pen*, Methodist DeBakey Cardiovascular Journal, US

## Abstract

Truman Capote (1924–1984) was a fascinating, entertaining, and much ballyhooed American character who came of age in the mid-Twentieth Century. Some would say he led a tragic life. Often described as a notable novelist, he was more generally a polymath dabbling in nonfiction between his parties. He also was a screenwriter, playwright, actor, and short-story writer. His literary classics include the novella *Breakfast at Tiffany’s* (1958, movie 1961) and true crime nonfiction “novel” (as Capote described it) *In Cold Blood* (1965, movie 1967). These two efforts became his most famous. But what about his poetry? Was this one of his creative passions?

## Plea From the Darkness

The sweet fragrance of spring’s perfume
Drifts to me out of the darkness,
And the soft sound of naked feet on the grass
Tells me the glories of living.
But here in the dark, vainly I search
For the pleasures of rustic Autumn,
For the sight of red and green and lavender,
And the brilliant sparkle of claret-colored wines.

Here in darkness—in this my world,
That only the brazen rhythm
And the mystenes of scene may penetrate—
I float aimlessly, like a curl of angel’s hair.
My heart beats wishingly, hopefully.
For the sight of a red leaf’s mad swirl to earth
And for the beauties of flesh:
For Spring has come—and I am Blind.

Truman Capote

Reprinted with permission from the Truman Capote Estate.

## Commentary

The Poetry Foundation is the go-to place for all things poetry.^1^ Their purpose is to recognize the “power of words to transform lives.” Specifically, the Foundation “amplifies poetry and celebrates poets by fostering spaces for all to create, experience, and share poetry.” It emerged from the Modern Poetry Foundation founded in 1941. Its programs include *Poetry Magazine*, which began in 1912 and featured luminaries such as T.S. Eliot, Ezra Pound, William Carlos Williams, Carl Sandburg, Wallace Stevens, and Marianne Moore, along with thousands having lesser fame.

If Truman Capote had any serious poetic output, he surely would have had his work represented on the Foundation’s website, as it is poetry’s equivalent of the “Oxford Dictionary.” With over 40,000 poems written by poets past and present, Capote would certainly be there—if not for the quality of his poems then for the notoriety of his personality. But there are no poems by Truman Capote at the Poetry Foundation website or in their other digital offerings. The Library of Congress index lists 371 reference items by Capote, about Capote, or critiques of his oeuvre. Where, then, did *Plea From the Darkness* appear?^2^

Truman Capote was born Truman Streckfus Persons in New Orleans on September 30, 1925.^3,4^ He had a difficult early life, with his parents divorcing. He spent long periods away from his mother, whose distant relatives raised him. He changed his name in 1935 to Truman Garcia Capote with “Capote” the name of his stepfather. He was a lonely but precocious child, teaching himself to read and write before starting school, with writings that go back to his pre-teen years.

**Figure:**
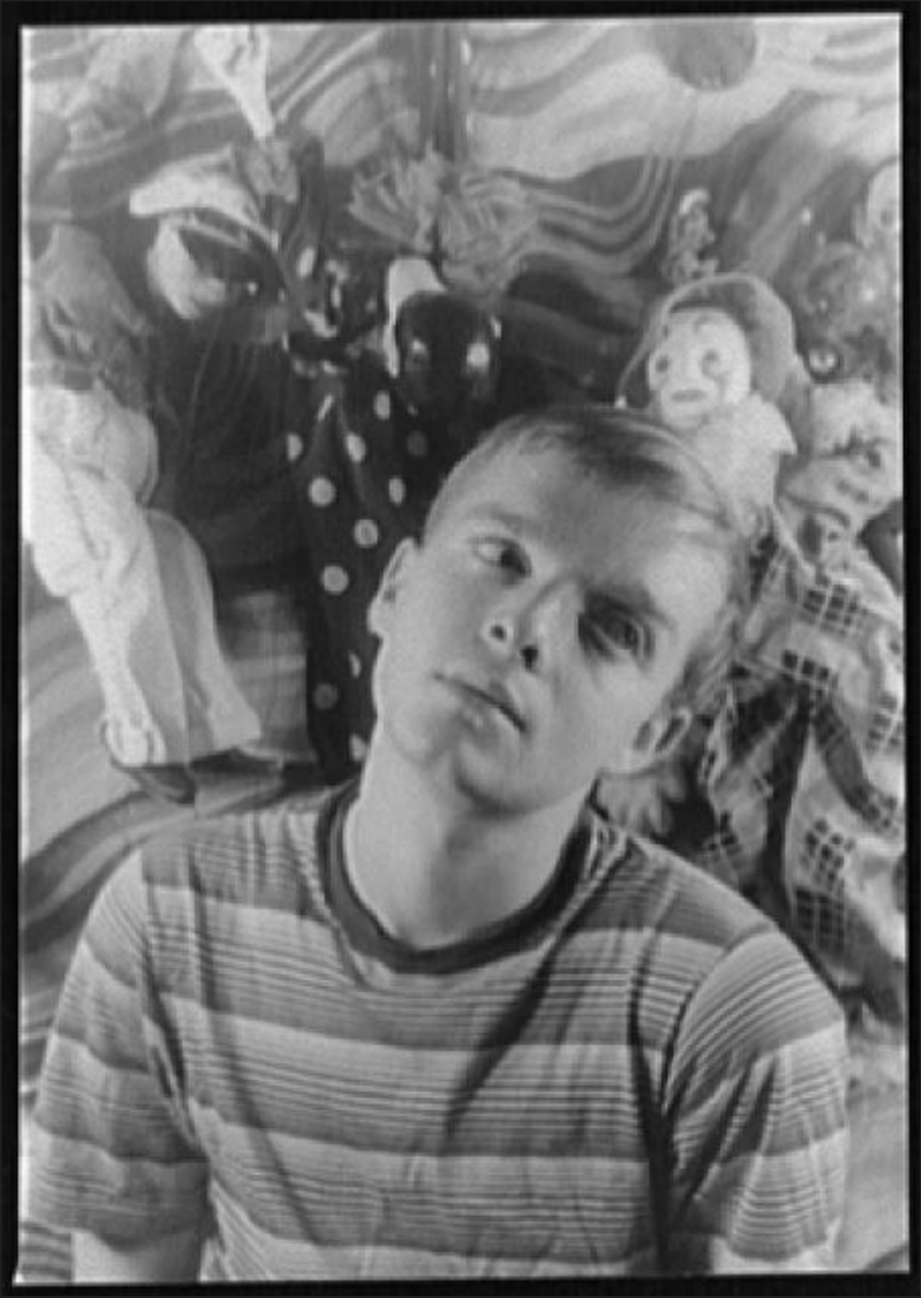
Truman Capote as a young man, in front of puppets and patterned backdrop. Library of Congress, Prints & Photographs Division, Carl Van Vechten Collection [reproduction number, eg, LC-USZ62-54231].

After his stepfather was convicted of embezzlement, the family was forced to leave a New York City Park Avenue residence and move to Greenwich, Connecticut, where he attended Greenwich High School. It was there that Capote really began his literary career. He wrote for both the school newspaper and its literary journal, *The Green Wich* (obviously a pun). It was in the school archives that his poetry, including *Plea From the Darkness*, was discovered.

The poems seem focused on moral conflict and longings. They use simple, nature-based imagery and are characteristic, in my view, of a struggling teenager confronting the challenge of maturation. Other than these early poems, there seem to be no others to study. Capote, in his prodigious literary output, placed his talent in different arenas.

One of Capote’s two most notable works was *Breakfast at Tiffany’s: A short novel and three stories* (1958). The novel, and subsequent 1961 movie, was about the character Holly Golightly and her careless spontaneity and carousing young adult years. But my attraction and link to Truman Capote was *In Cold Blood*, both the book in 1965 and the movie in 1967.^5^ The book, and later the movie, was largely a nonfiction drama, but Capote did play fast and loose with some of his recounting of the horrific mass murder of the Holcombe, Kansas, Clutter family in 1959.

The subsequent execution of the two perpetrators at Leavenworth Federal Penitentiary in 1965 fascinated Capote, who spent five years exploring the story and writing a magnificent lyrical, almost poetic, rendition of the awful story of Bonnie (Fox), Herbert Clutter, and two of their children, teenagers Nancy and Kenyon. The Clutters had two other daughters who lived away and survived: oldest child Eveanna (named after my great grandmother and living with her husband in Chicago), and next oldest, Beverly, who was studying nursing in Kansas City the night of the massacre.

My father was the first cousin of Bonnie Fox and my family, despite living in the San Francisco Bay Area at the time, was captured by the tragedy. That was my connection to Truman Capote. Being interested in literature during high school, and then attending as an undergraduate the University of Kansas, I devoured Capote’s story of the Kansas crime and became interested in his other works and the question of him being a poet. He entertained me with his bizarre antics and some of his works, particularly *In Cold Blood*, because of my connection to the Clutter family.

Truman Capote was a fascinating character exposed by his writings and society doings. He was in and out of a variety of drug rehabilitation clinics and struggled with alcoholism and other psychiatric issues. He died in Los Angeles on August 25, 1984, at the young age of 59, with the primary cause being liver failure and multiple drug intoxication. Though leaving much intriguing work, unfortunately, there is not much poetry.
